# A Systematic Review and Meta-Analysis of Sodium-Glucose Cotransporter 2 (SGLT-2) Inhibitors and Their Impact on the Management of Heart Failure

**DOI:** 10.7759/cureus.75802

**Published:** 2024-12-16

**Authors:** Nestor Lemos Ferreira, Abiodun Bamidele Adelowo, Zahid Khan

**Affiliations:** 1 Cardiology, University of South Wales, Wales, GBR; 2 Cardiology and Preventative Cardiovascular Medicine, University of South Wales, Wales, GBR; 3 Acute Medicine, Mid and South Essex NHS Foundation Trust, Southend-on-Sea, GBR; 4 Cardiology, Bart’s Heart Centre, London, GBR; 5 Cardiology and General Medicine, Barking, Havering and Redbridge University Hospitals NHS Trust, London, GBR; 6 Cardiology, Royal Free Hospital, London, GBR

**Keywords:** heart failure hospitalization, heart failure management programmes, heart failure prognosis, heart failure with preserved ejection fraction, heart failure with reduced ejection fraction, major adverse cardiovascular events (mace), preferred reporting items for systematic reviews and meta-analyses(prisma), remote monitoring of heart failure, sodium-glucose cotransporter-2 (sglt2) inhibitors, systolic heart failure

## Abstract

Heart failure (HF) is a life-threatening condition with severe incapacitating consequences. Many body organs and systems may be affected, which may also hinder the quality of life and finances at the individual and societal levels. Sodium-glucose cotransporter-2 inhibitors (SGLT2i) have also emerged as potentially useful drugs in the HF domain and other medical fields, in addition to their glucose-lowering effect. This systematic review followed the Preferred Reporting Items for Systematic Reviews and Meta-Analyses (PRISMA) guidelines, and the authors searched Google Scholar, PubMed, and Scopus websites for SGLT2i and SGLT2i-related terms and their impact on HF events, major adverse cardiovascular events (MACEs), renal composite outcomes, and improvement in the Kansas City Cardiomyopathy Questionnaire (KCCQ) scores, involving human adult populations. Two reviewers conducted the literature search, and disagreements were resolved through mutual consensus and input from a third reviewer. A literature search was conducted from 1st February to 20th February 2024. We included studies published after 2018 to focus only on the latest advancements. Randomized controlled trials, observational studies, or systematic reviews of these studies were included in our study. Of the 44 initial articles identified, only 14 met the inclusion and exclusion criteria. The outcomes revealed the superiority of SGLT2i therapeutics over placebo in all four domains mentioned above. A total of 234,509 patients from 11 papers with moderate heterogeneity (P = 0.07; I2 = 42%) evaluating the effect of SGLT2i in comparison to placebo on HF events were considered; of these, 128,477 patients received the intervention drug, and 106,032 individuals were assigned to the control group. The absolute numbers of HF events were 6845 and 8877, respectively. The study showed an overall benefit of SGLT2i in patients with heart failure due to their ability to major adverse cardiovascular events (MACE) in comparison to placebo (OR: 0.92; 95% CI: 0.89-0.96; P < 0.00001). This systematic review confirmed previous findings related to the use of SGLT2i as adjunctive therapy for HF and amelioration of KCCQ scores and as a protective agent against MACE and renal impairment progression.

## Introduction and background

Heart failure (HF) is a complex and life-threatening condition that affects between 1% and 2% of the world’s population and has rampant estimates for the next decades and occurs as a result of a combination of other medical ailments, such as diabetes mellitus, hypertension, chronic kidney disease, ischemic heart disease and unhealthy lifestyles [1,2]. HF is associated with significant financial costs both at the individual and societal levels, such as loss of revenues, early ill-health retirement, a heavy burden for healthcare systems, and alarming rising trends worldwide, especially in low- and middle-income nations associated with the emergent prevalence of non-communicable diseases [3]. Therefore, there is a need for novel strategies in managing this complex disease to reduce the morbidity and mortality associated with this disease [4].

Apart from the non-pharmacological HF approach, which includes lifestyle modification strategies to address underlying causes like obesity, hypertension (HTN), and type 2 diabetes mellitus (T2DM), as well as the use of traditional drugs, namely angiotensin-converting enzyme inhibitors (ACEIs), angiotensin-II receptor blockers (ARBs), beta-blockers (BBs), loop and thiazide diuretics, mineralocorticoid receptor blockers (MRBs) and costly cardiac procedures such as the implantable cardioverter-defibrillator device (ICD) and heart transplantation, in recent years, sodium-glucose cotransporter-2 inhibitors (SGLT2i) have been added by prominent medical societies worldwide, like the European Society of Cardiology (ESC) and the American Heart Association (AHA), to the HF therapeutic arsenal, given their positive outcomes in terms of reduced cardiovascular (CV) morbidity and mortality and HF hospitalizations, besides their glucose-lowering property to treat T2DM while binding to the SGLT2 molecule in the proximal convoluted tubule, thus impeding glucose reabsorption and consequently promoting glycosuria. These unexpected findings associated with SGLT2i and CV health were discovered after the US Food and Drug Administration (FDA) required evidence of CV safety involving the intake of new hypoglycemic agents [4-9]. Additionally, some evidence has also been attributed to them as likely retinal protective agents [10] and may also offer renal protection [11], thus placing them into a category of pleiotropic agents, as some authors say [12,13].

The primary aim of the present study was to assess the effect of SGLT2i therapy on HF events and major adverse cardiovascular events (MACEs). The secondary objective was to verify whether these drugs influence renal function laboratory findings or clinical presentation, as well as the assessment of the Kansas City Cardiomyopathy Questionnaire (KCCQ scores). There are two versions of the KCCQ score routinely used in clinical practice, with the original questionnaire consisting of 23 items and the modified version consisting of 12 items, known as (KCCQ-23) and (KCCQ-12), respectively. Both these scores include questions to assess symptom frequency, social limitations, physical limitations, and quality of life domains. Patients with lower KCCQ scores have poor health, which in turn is associated with increased all-cause mortality and HF-related hospitalization in hospitals.

## Review

This systematic review followed the 2020 PRISMA guidelines and PICO statement (Table 1).

**Table 1:** Population, intervention, comparison, and outcome (PICO) framework. MACE: major adverse cardiovascular events, HF: heart failure, SGLT2i: sodium-glucose co-transporter-2 inhibitors, HFrEF: heart failure with reduced ejection fraction, HFpEF: heart failure with preserved ejection fraction, T2DM: type 2 diabetes mellitus, KCCQ score: Kansas City Cardiomyopathy Questionnaire.

Variable	Definition
Population	Humans older than 18 years old are affected by all types of HF (HFrEF, HFpEF, and HFmrEF) regardless of T2DM status
Intervention	The administration of SGLT2i (canagliflozin, empagliflozin, dapagliflozin, ertugliflozin, and sotagliflozin)
Comparison/control	The control group was placebo-controlled
Outcome	Reduction of HF events and MACE, as well as evidence of renal protection and changes in KCCQ scores following SGLT2i therapy

Data source and search strategy

Two independent authors searched Google Scholar, PubMed, and Scopus, using the following terms: ‘sodium-glucose transporter 2 inhibitors/SGLT2i’, 'canagliflozin', 'empagliflozin', 'dapagliflozin', 'ertugliflozin', ‘sotagliflozin,' 'SGLT2i in HF management’. Any discrepancies were resolved through agreement and with input from a third reviewer. Moreover, database filters that included only English-language written articles, RCTs, meta-analyses, and systematic reviews from 2018 onwards were also considered. Studies published prior to 2018 were excluded to focus on the latest research data.

Study selection and eligibility criteria

As a result, 44 articles were found, which were then exported to the free web tool Rayyan to resolve duplications. Subsequently, all 41 remaining papers were screened and assessed for eligibility; however, only 14 were ultimately selected after full compliance with the inclusion and exclusion criteria. The Rayyan software was used collaboratively to exclude duplicate articles.

Statistical analysis

The Cochrane Review Manager (RevMan) 5.3 version (The Cochrane Collaboration, Copenhagen) and STATA 12.0 (StataCorp LP, College Station, TX) for meta-analysis were used to carry out the statistical analyses using pooled ORs, 95% CIs, the Mantel-Haenszel equation, and the random-effects model to compare the intervention (SGLT2i) and control (placebo) groups. The authors also used six domains of the Cochrane risk of bias tool to evaluate the quality of the selected studies: random sequence generation (selection bias), allocation concealment (selection bias), blinding of participants and personnel (performance bias), blinding of outcome assessment (detection bias), incomplete outcome data (attrition bias), and selective reporting (reporting bias). To check for publication bias, their respective funnel plots were also displayed after each forest plot related to the analyzed results.

**Figure 1:**
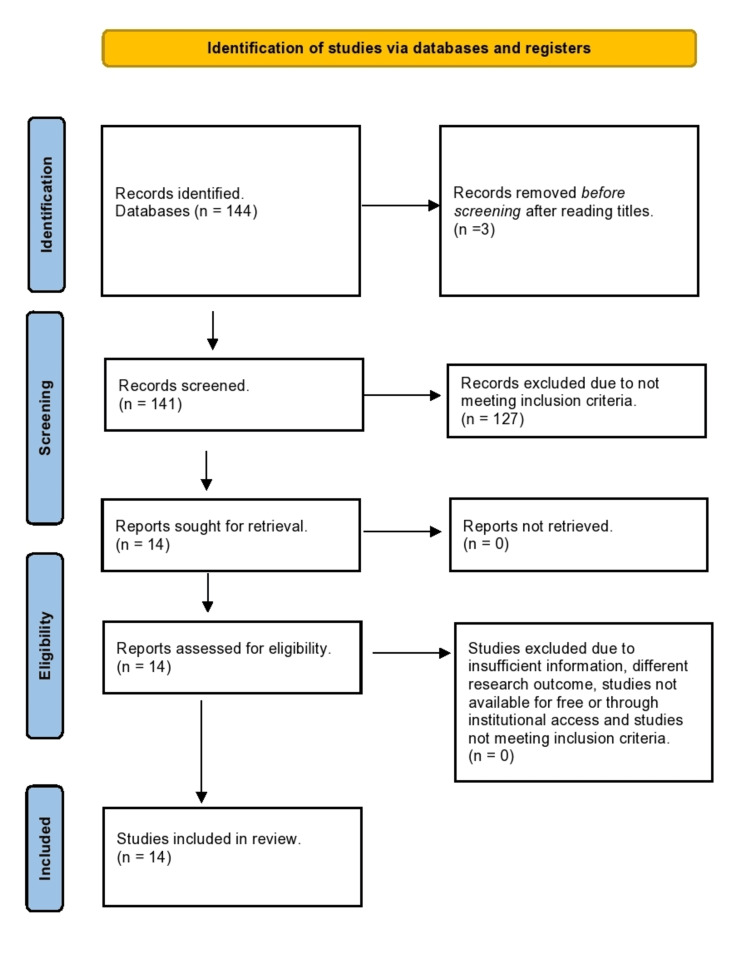
PRISMA flow diagram of literature search. PRISMA: Preferred Reporting Items for Systematic Reviews and Meta-Analyses.

The exclusion criteria were animal studies, pediatric population, reviews assessing only T2DM, reviews without focus on HF, studies without primary or secondary HF endpoints, research mentioning other SGLT2i than canagliflozin, empagliflozin, dapagliflozin, ertugliflozin, sotagliflozin, such as ipragliflozin, tofogliflozin, sergliflozin, remogliflozin, or luseogliflozin; research comparing SGLT2i to other hypoglycemic agents, for example, glucagon-like peptide 1 and metformin, languages other than English were not considered in this review, such as German and French, due to intellectual property reading restrictions, non-randomized interventional studies, case reports, qualitative research, letters to the editor, commentaries, conference proceedings, grey literature, opinions as well as policy papers and case series were not considered for this review. The keywords used for literature search are presented in Table 2, and the PRISMA flow chart is presented in Figure 1.

**Table 2:** Strategy search and keywords used. SGLT2i: sodium-glucose co-transporter-2 inhibitors, HF: heart failure.

Sodium-glucose transporter 2 inhibitors	SGLT2i in HF management
SGLT2i	Canaglifozin
Empaglifozin	Dapaglifozin
Ertuglifozin	Sotaglifozin

Results

The primary endpoints were HF events such as emergency department visits with HF, hospitalization for HF (HHF, either first or readmission, including death after HF worsening), and MACE, defined as CV death, MI, or ischemic stroke. The secondary endpoints were renal composite outcomes, defined as death due to nephropathy failure, renal replacement therapy (dialysis), or doubling of serum creatinine and no improvement in KCCQ scores. A total of 239,286 patients were included in the study, of which 130,866 were in the intervention group and 108,420 were in the placebo-controlled group. Table 3 provides a detailed summary of the characteristics of the studies included. There was variability based on the number of patients, ethnic variation, and selection of SGLT2i in these studies. Hence, a random effect model was used.

**Table 3:** Characteristics of the selected studies. RCT: randomized controlled trial, CV: cardiovascular, HF: heart failure, MACE: major adverse cardiovascular events, HHF: hospitalization for HF,T2DM: type 2 diabetes mellitus, CKD: chronic kidney disease, SD: standard deviation, KCCQ-CS: Kansas City Cardiomyopathy Questionnaire-clinical summary score, KCCQ-OS: Kansas City Cardiomyopathy Questionnaire-overall summary, KCCQ-PL: Kansas City Cardiomyopathy Questionnaire-physical limitation, KCCQ-TSS: Kansas City Cardiomyopathy Questionnaire-total symptoms, HFpEF: heart failure with preserved ejection fraction, HFrEF: heart failure with reduced ejection fraction, LVEDV: left ventricular end diastolic volume, LVESV: left ventricular end systolic volume, QoL: quality of life, EEA: European Economic Area, MI: myocardial infarction, ASCVD: atherosclerotic cardiovascular disease, CKD: chronic kidney disease, CREDENCE*: Canagliflozin and Renal Endpoints in Diabetes with Established Nephropathy Clinical Evaluation, CV: cardiovascular, DAPA-CKD: dapagliflozin in patients with kidney disease, with and without heart failure, DELIVER: dapagliflozin evaluation to improve the lives of patients with preserved ejection fraction heart failure, EMBRACE-HF: empagliflozin effects on pulmonary artery pressure in patients with heart failure, EMPA-TROPISM: empagliflozin in non-diabetic patients with heart failure and reduced ejection fraction, EMPEROR-preserved: Empagliflozin Outcome Trial in patients with chronic heart failure with preserved ejection fraction), EMPULSE: empagliflozin in patients hospitalized for acute heart failure, PRESERVED-HF: effects of dapagliflozin on biomarkers, symptoms and functional status in patients with preserved ejection fraction heart failure, VERTIS CV: eValuation of ertugliflozin efficacy and safety cardiovascular outcomes, QoL: quality of life.

Selected studies	Type of research	Participants statistics	Participants age	N SGLT2i	N control	Cohort	Type of intervention	Some of the study outcomes
Bhattarai et al., 2022 [14]	Meta-analysis of RCTs	28,809 Men	16,793 were younger than 65 years old	N = 39,053	N = 32,500	Participants with history of ASCVD or risk factors for ASCVD, T2DM, or HF	Canagliflozin	CV death or HHF
		15,655 Women					Dapagliflozin	
		Ethnicity					Empagliflozin	
		79.43% White	17,087 were older than 65 years old				Ertugliflozin	
		25.57% Asian					Sotagliflozin	
		19.14% Black					Placebo	
		14.35% Other						
Cosentino et al., 2020 [15]	Double-blind RCT (VERTIS CV trial)	5767 Men	Mean age >60 years old	N = 5499	N = 2747	T2DM patients with history of ASCVD	OD 5 mg Ertugliflozin	HHF and composite HHF/CV death
		2479 Women					OD 15 mg Ertugliflozin	
		Ethnicity					Placebo	
		Unavailable data						
Filippatos et al., 2022 [16]	RCT (secondary analysis of the EMPEROR-preserved trial)	3312 Men	Diabetic patients: 70.9 ± 9.0	N = 2997	N = 2991	HF patients with or without T2DM	OD 10 mg Empagliflozin	Primary and secondary HF and renal endpoints
		2676 Women						
		Ethnicity						
		75.85% White	Non-diabetic patients: 72.8 ± 9.7				Placebo	
		4.30% Black/African American						
		13.76% Asian						
		6% Other						
Kommu, 2014 [17]	Systematic review and meta-analysis of RCTs	6709 Men	Mean age >60 years old	N = 5316	N = 5322	Non-diabetic patients with HF	OD 10 mg Dapagliflozin	Composite of the first episode of worsening HF and CV death
		3929 Women						
		Ethnicity						
		72.18% White					OD 10 mg Empagliflozin	
		19.33% Asian						
		3.92% Black/African American					Placebo	
		4.57% Other						
McMurray et al., 2021 [18]	RCT (DAPA-CKD trial)	2879 Men	No HF patients: mean age 61.4 years	N = 2152	N = 2152	CKD patients with and without HF	OD 10 mg Dapagliflozin	End-stage kidney disease, composite CV death, HHF
		1425 Women						
		Ethnicity						
		53.20% White	HF patients: mean age 65.3 years					
		4.43% Black/African American					Placebo	
		34.08% Asian						
		8.29% Other						
Nassif et al., 2021 [19]	RCT (EMBRACE-HF trial)	41 Men	Empagliflozin: mean age ± SD 69.5 ± 12.0	N = 33	N = 32	HF patients (regardless of EF, with or without T2DM)	OD 10 mg Empagliflozin	Changes in the KCCQ-CS and KCCQ-OS
		24 Women						
		Ethnicity	Placebo mean age ± SD, 62.9 ± 13.3				Placebo	
		77% White						
		23% Black						
Nassif et al, 2021 [20]	RCT (PRESERVED-HF trial)	140 Men	Dapagliflozin: Mean age 69 years	N = 162	N = 162	Patients with HFpEF	Dapagliflozin	Changes in the KCCQ-CS, KCCQ-TS, KCCQ-PL, and KCCQ-CS scores
		184 Women						
		Ethnicity	Placebo: Mean age 71 years				Placebo	
		67% White						
		33% African American						
Pandey et al., 2022 [21]	Meta-analysis of RCTs	10,594 Men	Mean age >60 years old	N = 7841	N = 7843	HF patients with either HFrEF or HFpEF	Dapagliflozin	The composite of HHF and CV death and renal composite outcomes
		5090 Women						
		Ethnicity					Empagliflozin	
		74.23% White						
		16.55% Asian					Sotagliflozin	
		5.04% Black					Placebo	
		4.18% Other						
Peikert et al., 2022 [22]	Double-blind RCT (DELIVER trial)	3515 Men	40 to 99 years old	N = 3131	N = 3132	HF patients regardless of DM status	OD 10 mg Dapagliflozin	Composite of worsening HF events or CV death, change from baseline in the KCCQ-TSS score.
		2747 Women						
		Ethnicity						
		70.87% White						
		20.34% Asian						
		2.53% Black					Placebo	
		3.01% American Indian or Alaska Native						
		3.25 Other						
Rasalam et al., 2021 [23]	Systematic review	37,379 Men	Mean age >60 years old	N = 31,001	N = 24,442	T2DM patients with either CKD or high CV risk and HFrEF patients with or without T2DM	Canagliflozin	HHF
		18,056 Women					Dapagliflozin	
		Ethnicity					Empagliflozin	
		77.22% White					Ertugliflozin	
		18.38% Asian					Placebo	
		4.03% Black						
		0.37% Other						
Santos Galego et al., 2021 [24]	Double-blind RCT (EMPA-TROPISM trial)	54 Men	People whose ages were <65 years old and >65 years old	N = 42	N = 42	Non-diabetic HFrEF patients	OD 10 mg Empagliflozin	Changes in LVEDV and LVESV and QoL (KCCQ-12) among others
		30 Women						
		Ethnicity						
		27.38% White						
		50% Hispanic/Latino					Placebo	
		19.05% African American						
		3.57% Asian						
Voors et al., 2022 [25]	Double-blind RCT (EMPULSE trial)	351 Men	Empagliflozin: median 71 years	N = 265	N = 265	HF patients regardless of the EF	OD 10 mg Empagliflozin	All-cause mortality, HF events and change in KCCQ-TS score
		179 Women						
		Ethnicity						
		77.92% White	Placebo: median 70 years				Placebo	
		10.18% Black						
		10.75% Asian						
		1.15% Other						
Wada et al., 2022 [26]	Post-hoc analysis of the CREDENCE trial in EEA countries	2907 Men	EA participants: mean ± SD, 60.8 ± 9.1	N = 2202	N = 2199	T2DM patients with established nephropathy	OD 1000 mg Canagliflozin	The composite of HHF or CV death; the composite of MI, CV death, or stroke; CV death; HHF; all-cause mortality
		1494 Women						
		Ethnicity	Non-EEA participants: mean ± SD, 63.4 ± 9.2				Placebo	
		EEA participants: 100% Asian						
		Non-EEA participants: 7.19% Asian						
Zhang et al., 2020 [27]	Systematic review and meta-analysis of RCTs	37,582 Men	Mean age >60 years old	N = 31,172	N = 24,591	T2DM patients	OD Canagliflozin	HHF, MACE, and CV death
		18,173 Women					OD Dapagliflozin	
		Ethnicity					OD Empagliflozin	
		76.78% White					OD Ertugliflozin	
		14.77% Asian					Placebo	
		4.01% Black						
		4.44% other						

Effect of SGLT2i on heart failure (HF) events

**Figure 2:**
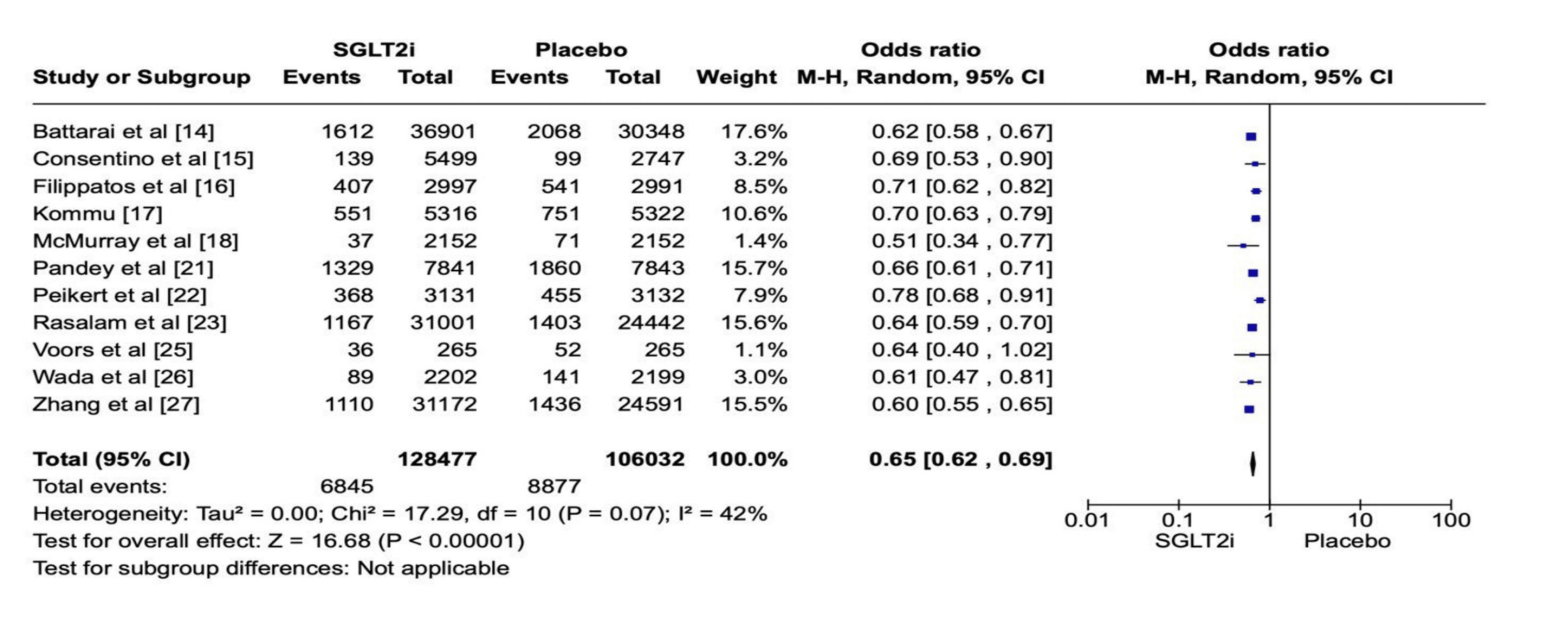
Forest plot of SGLT2i vs placebo related to HF events. SGLT2i: sodium-glucose co-transporter 2 (SGLT-2) inhibitors, HF: heart failure. Note: This image is the author's own creation.

**Figure 3:**
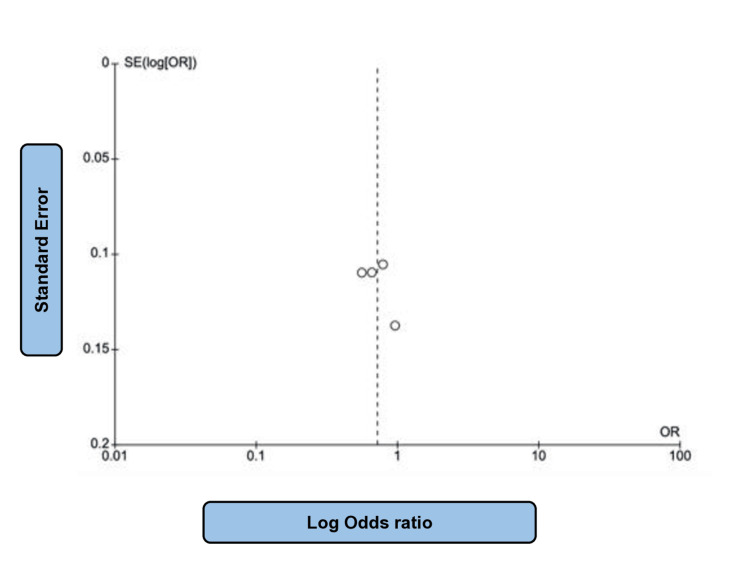
Funnel plot of SGLT2i studies related to HF events SGLT2i: sodium-glucose co-transporter 2 (SGLT-2) inhibitors, HF: heart failure. Note: This image is the author's own creation.

For such assessment, a total of 234,509 patients from eleven papers with moderate heterogeneity (P = 0.07; I2 = 42%) evaluating the effect of SGLT2i in comparison to placebo on HF events were taken into consideration [14-18,21-23,25-27], of which 128,477 patients received the intervention drug and 106,032 individuals were assigned into the control group. The absolute number of HF events was 6845 and 8877, respectively. Such findings allow one to infer that the administration of SGLT2i remarkably reduced HF events, that is, emergency department visits with HF and hospitalization for HF when compared to placebo (OR: 0.65; 95% CI: 0.62 to 0.69; P < 0.00001) (Figure 2). Figure 3 shows the corresponding funnel plot of the selected studies concerning HF events. These effects were noted in patients with both diabetic and non-diabetic patients across the studies.

Effect of SGLT2i on major adverse cardiovascular event (MACE)

**Figure 4:**
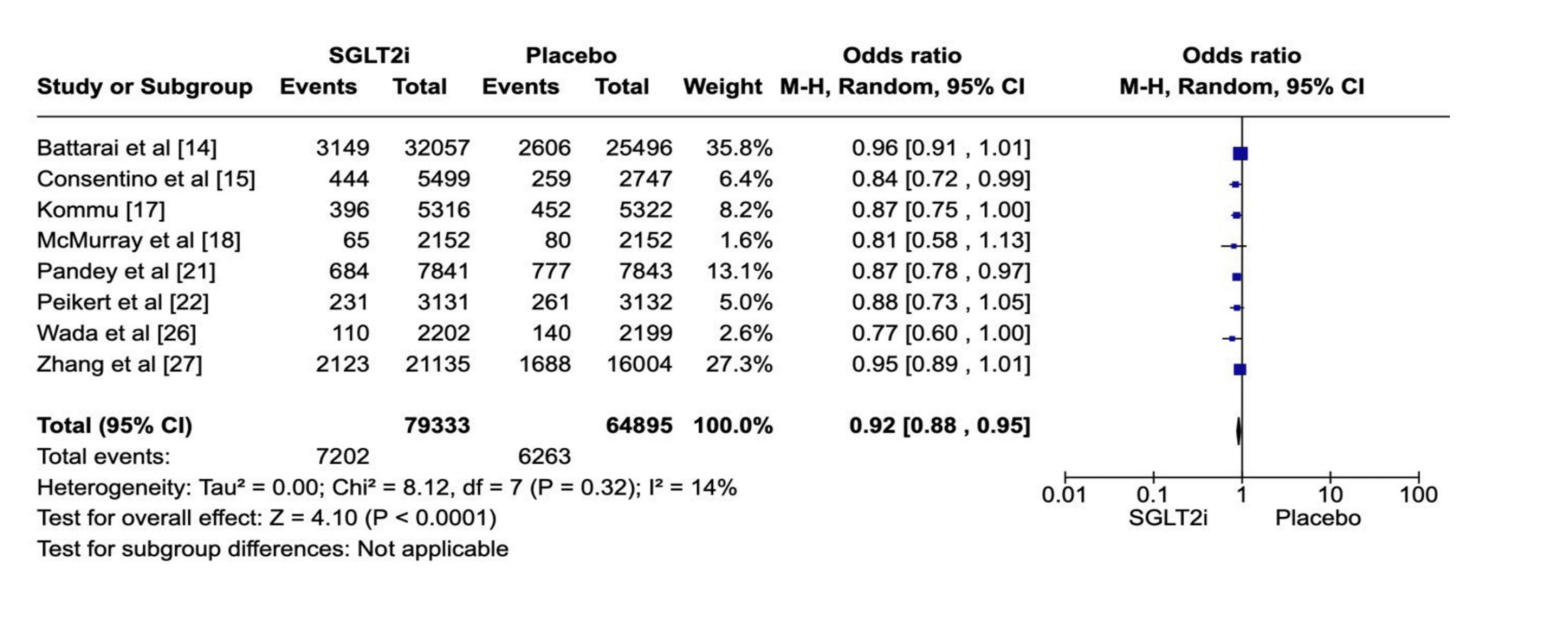
Forest plot of SGLT2i vs placebo regarding MACE SGLT2i: sodium-glucose co-transporter-2 inhibitors, MACE: major adverse cardiac events. Note: This image is the author's own creation.

**Figure 5:**
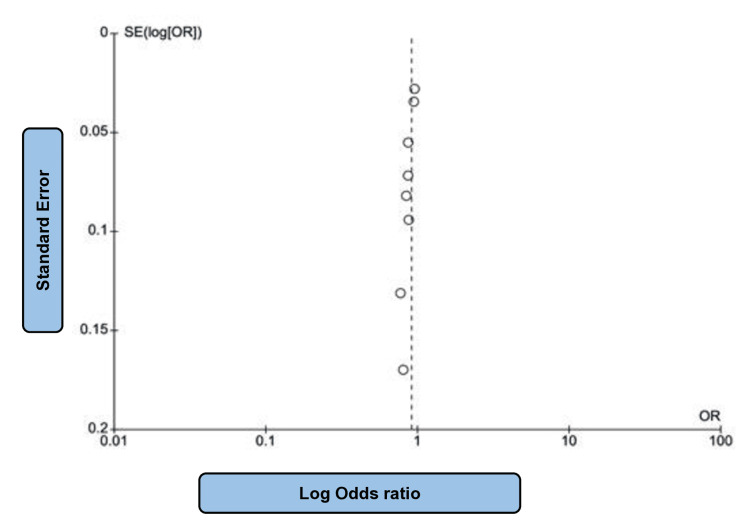
Funnel plot of SGLT2i studies regarding MACE. SGLT2i: sodium-glucose co-transporter-2 inhibitors, MACE: major adverse cardiac events. Note: This image is the author's own creation.

A total of 144,228 patients from eight investigations (studies 01, 02, 04, 05, 08, 09, 13, and 14) were randomly assigned to the intervention and control groups. While 79,333 patients received SGLT2i, 64,895 individuals received placebo; therefore, the absolute numbers of MACE were 7202 and 6254, respectively. Heterogeneity was low among the selected studies (P = 0.40; I2 = 14%), which shows consistency across these studies. It was found that there was an overall benefit of SGLT2i as they could reduce MACE in comparison to placebo (OR: 0.92; 95% CI: 0.89-0.96; P < 0.00001) (Figure 4). Figure 5 shows the corresponding funnel plots of the selected studies on MACE.

Effect of SGLT2i on composite renal outcomes

**Figure 6:**
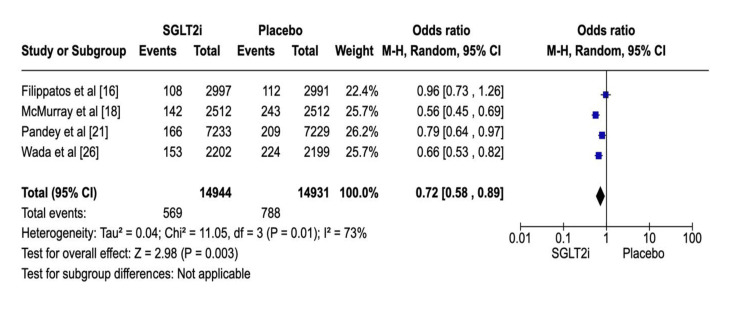
Forest plot of SGLT2i vs placebo concerning renal composite outcomes. SGLT2i: sodium-glucose co-transporter 2 inhibitors. Note: This image is the author's own creation.

**Figure 7:**
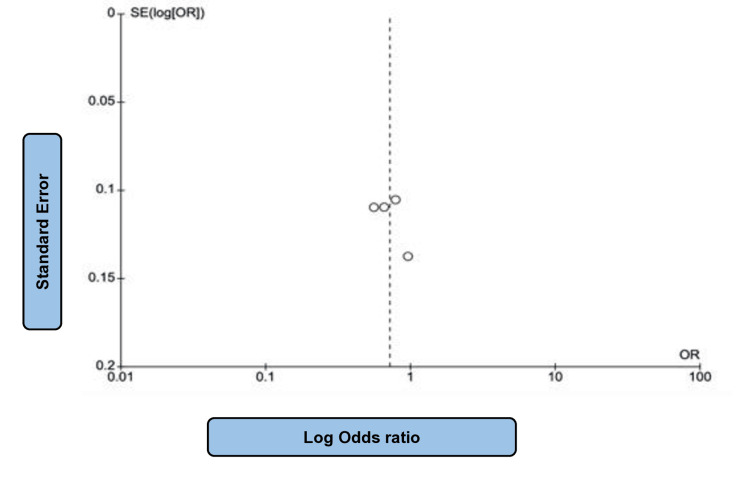
Funnel plot of SGLT2i research concerning renal composite outcomes. SGLT2i: sodium-glucose co-transporter-2 inhibitors. Note: This image is the author's own creation.

In this section, four studies [16,18,21,26], which included 29,875 patients, evaluated renal function. Of the 14,944 participants assigned to the SGLT2i group, 569 showed one of the following results: a two-fold increase in serum creatinine level, end-stage kidney disease (need for dialysis or renal transplantation), or renal death. Conversely, 788 patients in the control group showed similar results. Among these studies, the heterogeneity was high (P = 0.01; I2 = 73%). This high heterogeneity was likely due to population variation. Regarding such endpoints, composite renal outcome rates were lower following SGLT2i therapy (OR: 0.72; 95% CI: 0.58 to 0.89; P = 0.003) (Figure 6). Most studies showed that SGLT2i was effective in preventing the worsening of renal functions in patients with and without HF. Figure 7 shows the corresponding funnel plots of the selected studies related to renal composite outcomes.

Effect of SGLT2i on the improvement of Kansas City Cardiomyopathy Questionnaire (KCCQ) scores

**Figure 8:**
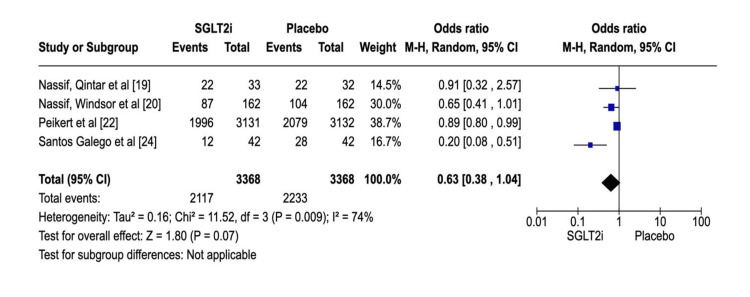
Forest plot of SGLT2i vs placebo evaluating no improvement in KCCQ scores. SGLT2i: sodium-glucose co-transporter-2 inhibitors, KCCQ: Kansas City Cardiomyopathy Questionnaire. Note: This image is the author's own creation.

**Figure 9:**
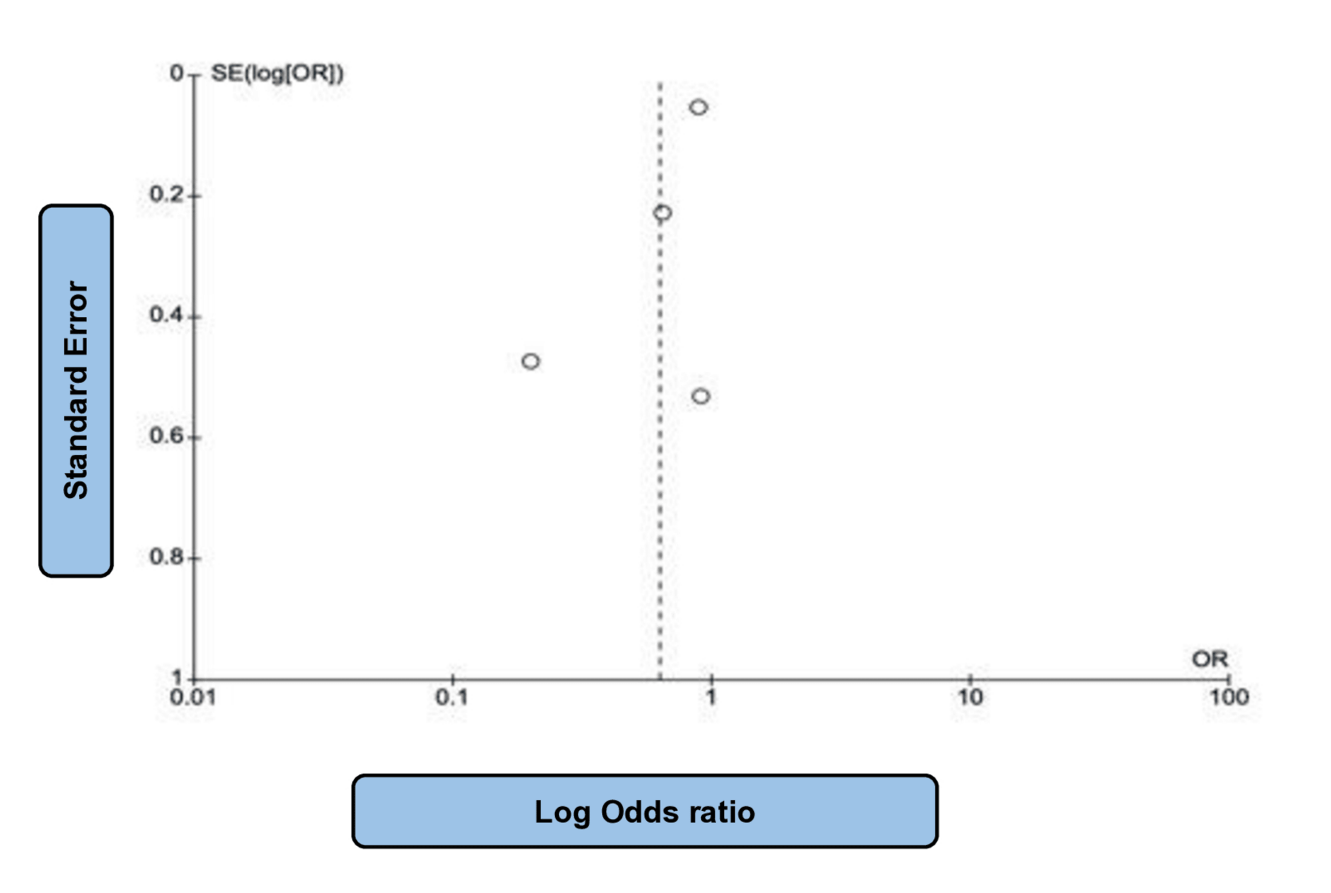
Funnel plot of SGLT2i analysis evaluating no improvement in KCCQ scores. SGLT2i: sodium-glucose co-transporter-2 inhibitors, KCCQ: Kansas City Cardiomyopathy Questionnaire. Note: This image is the author's own creation.

A total of 6736 patients from four studies [19,20,22,24] were randomly assigned to participate in this analysis, which considered their KCCQ scores. The intervention and control groups had an equivalent participant sum of 3368 individuals. In the SGLT2i group, 2117 reported no improvement in KCCQ scores, while 2233 displayed equal announcements. The heterogeneity was high (P = 0.009; I2 = 74%). The results showed a slight difference between the groups (OR: 0.63; 95% CI: 0.38-1.04; P = 0.07) (Figure 8), confirming the superiority of SGLT2i over placebo. Figure 9 shows the corresponding funnel plot of the selected studies regarding the improvement in KCCQ scores.

**Figure 10:**
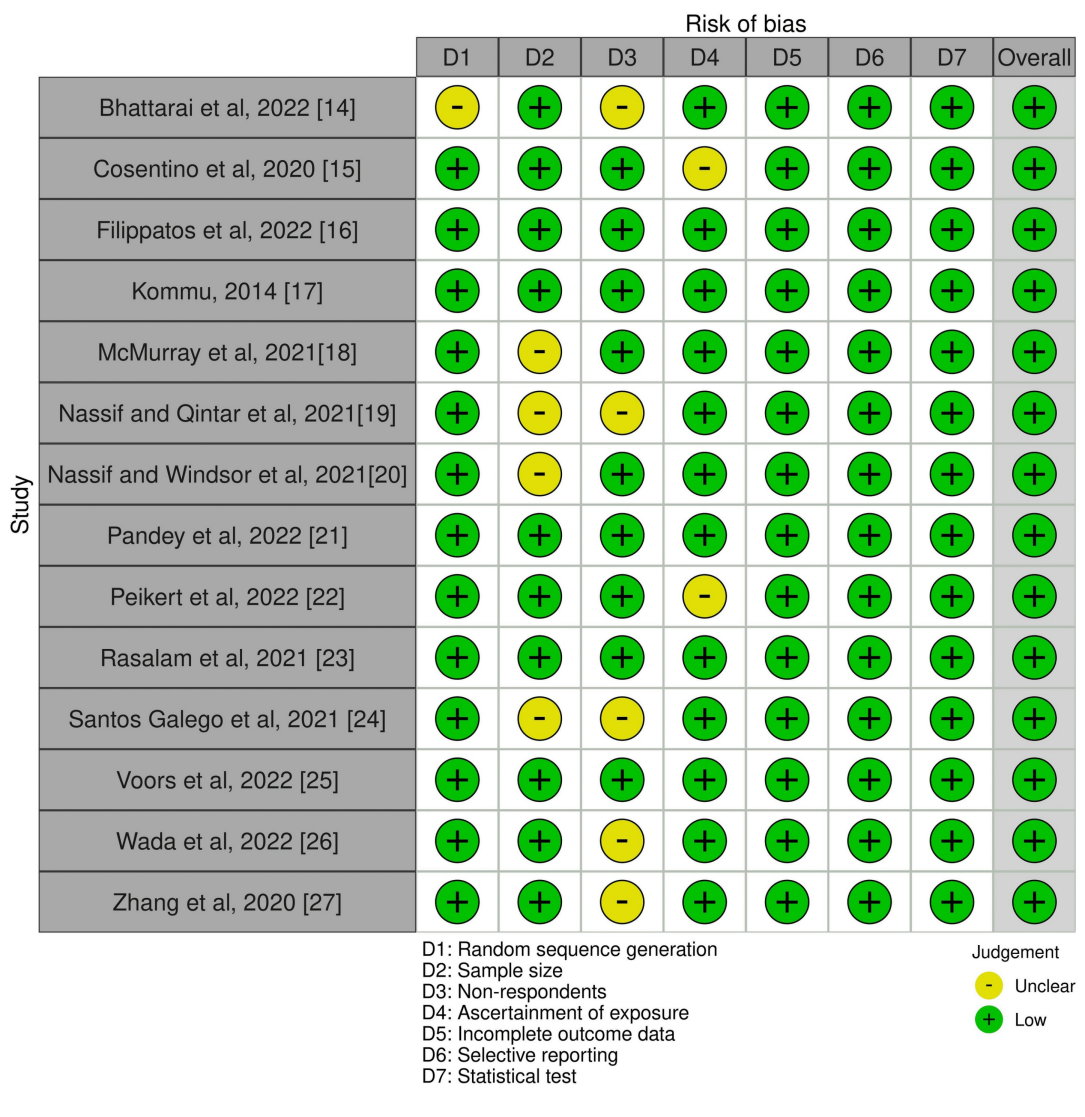
Risk of bias traffic light plot involving all the 14 selected studies in this systematic review. Note: This image is the author's own creation.

**Figure 11:**
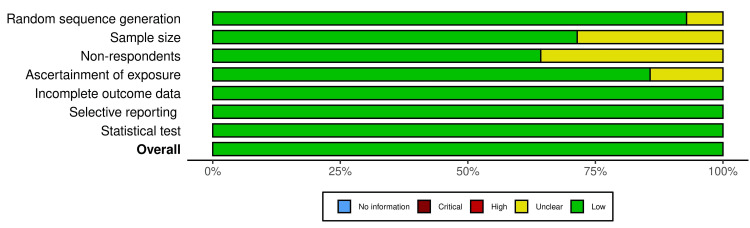
Summary plot for risk of bias assessment for the included studies. Note: This image is the author's own creation.

The risk of bias traffic light chart and risk assessment plots are presented in Figures 10, 11.

Discussion

More than 100 years after the recognition of SGLT2i predecessors and a better understanding of kidney physiology involving sodium-glucose co-transport, some of the first SLGT2i were approved for the management of T2DM in humans by prominent regulatory medical bodies. By 2015, canagliflozin, dapagliflozin, and empagliflozin had become part of the blood-glucose-lowering arsenal as supportive agents to be used either with biguanides or with insulin secretagogues [28]. Moreover, considering that T2DM is a risk factor for HF and CVD, it is of utmost importance to include these patients in CV safety analysis under SGLT2i use, as they were first launched as hypoglycemic agents. Their results showed superior protection against CV morbidity and mortality and HHF events [14,15,26,27]. However, further evidence indicates that HF patients have improved HF outcomes, regardless of DM status [16,17,19,21,24,25]. The EMPA-REG OUTCOME trial evaluated the impact of empagliflozin on CV morbidity and mortality. Their research involved a double-blind random assignment of 7020 high-risk for CVD T2DM adult patients who had received different doses of empagliflozin (10 or 25 mg daily) or placebo. The higher likelihood of CV events was defined as rest angina or recent heart attack in the past 60 days before the patients decided to take part in the survey, documented severe cardiac allograft vasculopathy towards the dominant left coronary artery or affecting at least two arterial blood vessels, confirmed atherosclerotic disease regarding constricted blood flow in one single artery, records of cerebral circulatory troubles (regardless of the etiology: either ischemic or hemorrhagic), and peripheral obliterative arteriopathy. By 2017, after the EMPA-REG OUTCOME cohort was released, it had already been established that empagliflozin could also contribute to the better management of HTN, reduction in body mass, and improvement in renal function. Therefore, Neal et al. further assessed another SGLT2i drug while conducting the Canagliflozin Cardiovascular Assessment Study (CANVAS)/CANVAS-Renal (CANVAS-R) double-blind, randomized trials to assess CV and nephrological endpoints following the administration of canagliflozin or placebo in patients with high CV risk, similar to the cluster studied by Zinman et al., two years before. Patients with HHF were considered for at least 12 hours of stay at the emergency unit and subsequent admission or discharge due to worsening of symptoms and refinement of CHF therapeutics in the EMPA-REG OUTCOME trial. A comparative analysis between the intervention and control groups in the EMPA-REG OUTCOME showed that empagliflozin reduced the risk of any cause of death and HHF by 32% and 35%, respectively [29].

The CANVAS/CANVAS-Renal (CANVAS-R) enrolled 10,142 participants, indicating remarkably fewer CV events along with HHF and lower exacerbation of renal parameters in favor of the intervention group when compared to placebo at the expense of increased chances of lower extremity dismemberment, namely phalanx and metatarsal amputations [30]. The analysis of safety outcomes was another important aspect of this study, and some trials involved in this systematic review were also named, such as genital and urinary tract infections, hypovolemia, acute kidney insufficiency, diabetic ketoacidosis, severe hypoglycemia, and amputation of lower extremities [16,18-20,25,26]. Although the frequency of adverse events is directly proportional to age, these drugs are considered safe even among frail elderly individuals over 75 years of age [22]. This study did not include the safety outcomes as a goal.

Further trials evaluating the role of SGLT2i in HF patients included the DECLARE-TIMI 58 trial, the CREDENCE trial, the DAPA-HF trial, the EMPEROR-reduced trial, the VERTIS CV trial, the SOLOIST-WHF trial, the SCORED trial, the EMPEROR-preserved trial, the CHIEF-HF trial, and the DELIVER trial. The role of dapagliflozin profile for CV protection was uncertain prior to the (DECLARE-TIMI 58) trial. To resolve this matter, Wiviott et al., by random assignment of 17,160 T2DM participants older than 40 years with precedent ASCVD or at greater possibility of evolving with such outcome, found, despite previous advantageous results from other contributors, no superior effect related to dapagliflozin on MACE, but significant benefits as to diminished HHF [31], in contrast to the reduced MACE occurrence found by Zhang et al. [27] and the present systematic review. The Canagliflozin and Renal Endpoints in Diabetes with Established Nephropathy Clinical Evaluation (CREDENCE trial) was another major study evaluating the efficacy of SGLT2i in HF patients, following the benefits demonstrated by previous studies. This study demonstrated the nephroprotective effects of the SGLT2i, which were validated later by other studies [16,18]. This study included 4401 patients; those assigned to the canagliflozin group also showed lower rates of CV death, heart attack, brain apoplexy, and HHF without evidence of increased adverse events, namely, amputation. Regarding the main endpoint, there were 34% fewer incidents of kidney impairment, which was attributed to a two-fold increase in serum creatinine levels and renal disease-related mortality. In contrast, [21] showed no statistically significant difference between the groups in terms of protection against kidney injury. Notably, the above-cited experiments were not the only ones conducted worldwide. Meanwhile, other researchers worldwide, surprised by such breakthrough discoveries, tried to elucidate whether those results would be similar in different subsets of patients affected by CHF, specifically in HFpEF, HFmrEF, and HFrEF [32]. 

In another phase 3 study, the Dapagliflozin and Prevention of Adverse Outcomes in Heart Failure (DAPA-HF trial), McMurray et al. carried out an investigation that included 4744 patients regardless of DM status allocated into the NYHA II, III, and IV classifications with EF no greater than 40% to dapagliflozin 10 mg OD and placebo groups. The primary outcomes in this study were heart failure hospitalization and intravenous diuretics requirement. Two thousand three hundred seventy-three and 2371 patients were randomized to dapagliflozin and placebo groups, respectively. Three hundred eighty-six and 502 patients had the primary outcome in the intervention and placebo groups, respectively. The frequency of hospital admission for heart failure and intravenous diuretics requirement was less in the dapagliflozin group compared to the placebo group. The risk of worsening heart failure and death from cardiovascular causes was lower in the dapagliflozin group, regardless of the presence or absence of diabetes [33]. The EMPEROR-reduced trial, on the other hand, enrolled 3730 patients who were randomly assigned to empagliflozin (1863 patients) or placebo (1867 patients) respectively. The median outcome follow-up period in this trial was 16 months compared to 18.6 months in the DAPA-HF trial. The primary outcome in this trial was similar to the DAPA-HF trial, which was hospitalization for worsening heart failure and death from cardiovascular causes. The findings from this trial demonstrated the efficacy of empagliflozin over placebo and showed reduced heart failure-related hospitalization rates and lower progression of CKD in the intervention group. These findings supported the findings from previous studies showing the efficacy of other SGLT2i in patients with heart failure. Similarly, patients in the empagliflozin group had a slower decline in the estimated glomerular filtration rate and lower progression of CKD compared to the placebo group. The risk of cardiovascular death was 18% lower in the DAPA-HF intervention group and 8% lower in the empagliflozin treatment group in this study as compared to placebo groups [34]. Patients in the empagliflozin group reported improved quality of life using a self-reported questionnaire (KCCQ) and the evaluation of LVEDV and LVESV as compared to patients without diabetes. These findings were similar to the DAPA-HF trial, showing improvement in the KCCQ score in patients receiving dapagliflozin as compared to the placebo group. 

Another Evaluation of Ertugliflozin Efficacy and Safety Cardiovascular Outcomes Trial (VERTIS CV), which was a multicentered double randomized controlled trial, enrolled 8246 patients. Five thousand four hundred ninety-three patients (11.9%) were randomized to receive the ertugliflozin, and 2745 patients were randomized to the placebo group. A major adverse cardiovascular outcome was noted in 653 of 5493 patients (11.9%) in the ertugliflozin group and 327 of 2745 patients (11.9%) in the placebo group. Hospitalization for heart failure or death from cardiovascular causes occurred in 444 of 5499 patients (8.1%) in the ertugliflozin group and 250 of 2747 patients (9.1%) in the placebo group. The study demonstrated the effectiveness of ertugliflozin exerts in reducing HHF and non-inferior MACE outcomes compared to placebo [35]. Another study in the same year showed diminished HHF and composite HHF/CV death in an ertugliflozin group compared to the placebo group. More urinary tract infections were reported in the ertugliflozin group compared to the placebo, and diabetic ketoacidosis occurred in seven patients (0.3%) and 12 patients (0.4%) who received the 5-mg and 15 mg doses of ertugliflozin as compared to placebo group where only two patients (0.1%) developed diabetic ketoacidosis. The** **Effect of Sotagliflozin on Cardiovascular Events in Patients with Type 2 Diabetes Post-Worsening Heart Failure (SOLOIST-WHF) trial evaluated the efficacy of Sotagliflozin on Cardiovascular Events in Patients with Type 2 diabetes post-worsening heart failure (SOLOIST-WHF) trial assessed the consequences of sotagliflozin intake (n = 608) in T2DM patients with acute health deterioration concerning CHF symptoms compared with placebo (n = 614). The composite endpoints in this study were hospitalization for heart failure and death from cardiovascular events. The study demonstrated the superiority of sotagliflozin in reducing HHF rates and deaths from cardiovascular events with lower events in the intervention group [36]. Side effects such as diarrhea and hypoglycemia were more common in the intervention group; however, hypotension and acute kidney injury were similar in both groups. 

The Effect of Sotagliflozin on Cardiovascular and Renal Events in Patients with Type 2 Diabetes and Moderate Renal Impairment Who Are at Cardiovascular Risk (The SCORED trial) evaluated the efficacy of sotagliflozin in reducing cardiovascular risk among T2DM patients with CKD. Bhatt et al. enrolled 10,584 patients in this study, with 5592 patients randomized to the sotagliflozin group and 5592 to the placebo group, respectively. This study supported the findings for SGLT2i from previous studies, and adverse effects such as diabetic ketoacidosis, hypovolaemia, diarrhea, and urinary tract infections were more common in the sotagliflozin group [37]. These findings were similar to those from previous studies, including the canaglifloz study by Neal et al. [37].

The following studies assessed the efficacy of SGLT2i in HFpEF patients. These studies assessed the efficacy of empagliflozin, canagliflozin, and dapagliflozin in this population; the results are briefly described below and were later confirmed by other researchers [18-21,25].

The Empagliflozin Outcome Trial in Patients with Chronic Heart Failure with Preserved Ejection Fraction (EMPEROR-preserved) enrolled 5988 patients with LVEF >40% classified as NYHA II to IV regardless of the diabetes status for assessment to evaluate the efficacy of SGLT2i. The primary endpoints in this study were similar to previous studies, such as cardiovascular death or hospitalization for heart failure. The empagliflozin arm showed a significant 29% reduced chance of HHF in comparison with the placebo. Empagliflozin was also associated with a reduction in cardiovascular death as compared to the placebo group, and these findings were consistent across the subgroups, irrespective of the status of diabetes [38]. The primary outcome event occurred in 511 of 2991 patients from the placebo group and 415 of 2997 patients from the empagliflozin group over a 26-month follow-up period. Some of the papers included in this review had comparable results when assessing dapagliflozin, empagliflozin, and sotagliflozin in patients with HFpEF [17,20-22], including patients who were hospitalized due to worsening HF and started empagliflozin before hospital discharge [25]. Adverse events such as genital and urinary tract infections and hypotension were more frequently observed in the empagliflozin group. 

The Canagliflozin: Impact on Health Status, Quality of Life, and Functional Status in Heart Failure (CHIEF-HF) remote trial carried out during the COVID-19 pandemic by Spertus et al. assessed canagliflozin (n = 222) or placebo (n = 226) administration in HF patients irrespective of their EF and the absence or presence of DM impact on the Kansas City Cardiomyopathy Questionnaire Total Symptom Score (KCCQ TSS), which was higher in the intervention group than in the placebo group [39]. This trial was stopped early due to a lack of funding by the sponsor. This trial differed from previous trials as it was a remote follow-up trial instead of an in-person one. The primary outcome, a change in the Kansas City Cardiomyopathy Questionnaire Total Symptom Score (KCCQ TSS) at 12 weeks, was 4.3 points in the canagliflozin group compared to the placebo group. This was observed in participants with HFrEF and HFpEF irrespective of diabetes status, demonstrating the effectiveness of canagliflozin in HF patients. Adverse events incidence was slightly higher in the canagliflozin group, although this difference was statistically not significant.

The Dapagliflozin Evaluation to Improve the Lives of Patients with Preserved Ejection Fraction Heart Failure (DELIVER) was another trial conducted to assess the efficacy of dapagliflozin in reducing HHF in patients with EF above 40% irrespective of their diabetes status. A total of 6263 randomly assigned patients received either 10 mg OD dapagliflozin (n = 3131) or placebo (n = 3132). The primary endpoints in this trial were HHF and death from cardiovascular events, which were reported as 16.4% vs 19.5% in the dapagliflozin vs placebo groups, respectively. There was no difference in the primary kidney endpoint based on the eGFR change in the dapagliflozin and placebo groups. The findings from this trial were consistent with the EMPEROR-preserved analysis, supporting the findings that dapagliflozin was a safe and meaningful therapy for patients with HF independent of EF and its coexistence with diabetes [40]. A brief summary of these trials is presented in Table 4.

**Table 4:** Previous landmark trials on SGLT2i and their favorable outcomes related to CV, HF events, slow progression of renal disease and improvement in KCCQ scores. SGLT2i: sodium-glucose co-transporter-2 inhibitors, KCCQ: Kansas City Cardiomyopathy Questionnaire, HF: heart failure, ASCVD: atherosclerotic cardiovascular disease, CANVAS-R: the Canagliflozin Cardiovascular Assessment Study-Renal, CHIEF-HF: Canagliflozin: Impact on Health Status, MACE: major adverse cardiovascular events, HFrEF: heart failure with reduced ejection fraction, HFpEF: heart failure with preserved ejection fraction, T2DM: type 2 diabetes mellitus, DM: diabetes mellitus, CREDENCE: canagliflozin and renal endpoints in diabetes with established nephropathy clinical evaluation, DAPA-HF: dapagliflozin and prevention of adverse outcomes in heart failure, DECLARE-TIMI 58: The Dapagliflozin Effect on Cardiovascular Events-Thrombolysis in Myocardial Infarction 58, DELIVER: dapagliflozin evaluation to improve the lives of patients with preserved ejection fraction heart failure, REG OUTCOME: Empagliflozin Cardiovascular Outcome Event Trial in Type 2 Diabetes Mellitus Patients-Removing Excess Glucose, EMPEROR-preserved: The Empagliflozin Outcome Trial in Patients with Chronic Heart Failure with Preserved Ejection Fraction, EMPEROR-reduced: Empagliflozin Outcome Trial in Patients with Chronic Heart Failure with Reduced Ejection Fraction, HHF: Hospitalizations for heart failure, HFrEF: heart failure with reduced ejection fraction, HFpEF: heart failure with preserved ejection fraction, KCCQ: Kansas City Cardiomyopathy Questionnaire, MACE: major adverse cardiovascular events, RCT: randomized controlled trials, SGLT2i: sodium-glucose co-transporter-2 inhibitors, SCORED: sotagliflozin on cardiovascular and renal events in patients with type 2 diabetes and moderate renal impairment who are at cardiovascular risk, SOLOIST-WHF: sotagliflozin on cardiovascular events in patients with type 2 diabetes post-worsening heart failure, VERTIS CV: evaluation of ertugliflozin efficacy and safety cardiovascular outcomes.

Remarkable previous trials (publication year)	Type of SGLT2i vs placebo	Population	SGLT2i benefits	SGLT2i disadvantages	Study relevance
EMPA-REG OUTCOME (2015) [29]	Empagliflozin	T2DM patients	Lower CV mortality and morbidity rates	Occurrence of genital infections	The first trial on SGLT2i and their protection against CV/HF events
			Lower HHF		
CANVAS/CANVAS-R (2017) [30]	Canagliflozin	T2DM patients with high CV risk	Lower CV events	Amputation of lower extremities	Assessment of renal outcomes
			Lower renal impairment and nephropathy-related deaths		
DECLARE-TIMI 58 (2018) [31]	Dapagliflozin	T2DM patients with previous ASCVD or at high risk of it	Lower CV deaths	No substantial protection against MACE	Lessen HHF rates
			Lower HHF	Development of genital infections	
CREDENCE (2019) [32]	Canagliflozin	T2DM patients with CKD	Lower risk of kidney failure	-	Similar rates of adverse events
			Lower CV events		
DAPA-HF (2019) [33]	Dapagliflozin	HFrEF patients with or without T2DM	Lower HF events	-	The first trial on HFrEF
			Lower CV deaths		SGLT2i benefits irrespective of DM status
					Similar rates of adverse events between groups
EMPEROR-reduced (2020) [34]	Empagliflozin	HFrEF patients with or without T2DM	Lower HHF	Cystitis	Additional evidence of SGLT2i efficacy in the HFrEF approach
			Lower CV deaths		
			Slower renal deterioration		
VERTIS CV (2020) [35]	Ertugliflozin	T2DM patients with ASCVD	Non-inferiority of ertugliflozin effect concerning MACE	The higher the ertugliflozin dose, the more frequent were amputations	Assessment of ertugliflozin
SOLOIST-WHF (2020) [36]	Sotagliflozin	T2DM patients and the first SGLT2i dose related to HHF	Lower HF events	Watery feces	Assessment of sotagliflozin
			Lower CV deaths	Alarming low glucose levels	SGLT2i introduction related to HHF
SCORED (2020) [37]	Sotagliflozin	T2DM patients with CKD	Lower HF events	Diabetic ketoacidosis	Assessment of sotagliflozin in respect of renal function
				Hypovolemia	
			Lower CV deaths	Watery feces	
				Urinary tract infections caused by fungi	
EMPEROR-preserved (2021) [38]	Empagliflozin	HFpEF patients with or without T2DM	Lower HHF	Cystitis	The first trial on HFpEF
				Hypotension	Benefits in favor of SGLT2i regardless of DM status
CHIEF-HF (2022) [39]	Canagliflozin	HFpEF, HFmrEF, and HFrEF with or without T2DM	Better improvement in KCCQ scores in the SGLT2i group	-	Remote clinical trial
					Assessment of KCCQ scores
					No adverse events related to SGLT2i use
DELIVER (2022) [40]	Dapagliflozin	HFmrEF or HFpEF patients with or without T2DM	Lower HF events	-	Use of KCCQ scores
			Lower CV deaths		More data on HFmrEF and HFpEF
					Similar rate of adverse events

The EMBRACE study by Nassif et al., despite its narrow sample, is a useful addition to the body of evidence supporting the use of SGLT2i in patients with HfpEF. This was one of the few studies on the variation in KCCQ scores and pulmonary artery (PA) pressure in patients with refractory HF classified into the New York Heart Association (NYHA) III or IV. Although empagliflozin showed no significant difference between the placebo and intervention groups in KCCQ scores, it could reduce PA pressure in the short term and appeared to be more notorious over time [19].

However, further work by Nassif et al., as they randomized a larger number of patients, depicted amelioration in KCCQ scores in HFpEF after 12 weeks of the dapagliflozin regimen in the PRESERVED-HF trial [20]. In this regard, EMPA-TROMPISM also evaluated the effects of empagliflozin consumption on KCCQ scores measured by symptom improvement over six months in HFrEF patients without DM, thus confirming the importance of SGLT2i in HF management [24]. Similar endpoints were also found by Voors et al. in the EMPULSE trial while considering hospitalized patients with decompensated HF [25]. The remarkable aspect of this study is that it may help clinicians and cardiologists in decision-making by prescribing SGLT2i before hospital discharge to prevent HF exacerbation. Moreover, in contrast to DAPA-HF research, which included only HFrEF patients irrespective of their T2DM condition [33], McMurray et al. assessed CKD patients without considering the presence or absence of HF in terms of renal and cardiovascular events, including HF hospitalization, after 10 mg dapagliflozin intake (DAPA-CKD trial). The promising broadened results not only matched the endpoints but also showed reduced rates of CKD-related death, regardless of DM or HF status [18].

In their post-hoc evaluation of the CREDENCE trial, Wada et al. assessed the influence of 100 mg canagliflozin intake on T2DM nephropathic patients in East Asian countries. Similar to the original research, this trial demonstrated that SGLT2i administration protects against renal and cardiovascular events [26].

Strengths

Considering all the above, this systematic review has satisfactorily contributed to evidence-based medicine (EBM) gold-standard practice. It has endorsed current trends in the medical literature concerning the benefits of SGLT2i in managing HF, that is, decreased emergency department visits with HF, first hospitalization for HF, or readmissions due to deteriorated clinical sets. The rates found in this systematic review were 5.3% and 8.4% in the SGLT2i and placebo groups, respectively. These outcomes represent 37% fewer events following SGLT2i administration than in the control group. With regard to MACE (CV death, MI, or stroke), eight selected studies showed significantly lower MACE rates in the intervention group than in the placebo group. However, favorable results associated with SGLT2i use in the adult human population were demonstrated in terms of preventing renal worsening or even nephropathy-related death while considering the four studies that were chosen for the analysis of this research’s secondary endpoints. Moreover, the superior outcomes associated with SGLT2i administration considered a wide range of patients irrespective of their history of ASCVD, age, DM status, EF values, ethnicity, sex, and chronic kidney disease. Most studies did not show any renal function deterioration in the SGLT2i groups compared to placebo. Further strengths of this systematic review are the large sample size and the inclusion of paramount RCTs, such as the VERTIS CV, DAPA-CKD, DELIVER, EMBRACE-HF, and EMPULSE trials, as well as other meta-analyses and systematic reviews in the investigation, contributing to a low-risk of bias scrutiny and its transparency regarding strict methodological process execution, thus making it reproducible and amplifying both internal and external validity.

Limitations

On the other hand, considering its limitations, one should consider the lack of data on safety outcomes to help better decision-making in clinical practice. Moreover, the scarcity of research on the effects of sotagliflozin and ertugliflozin, assessing the primary and secondary endpoints considered in this study, was another weakening spot, not to mention the exclusion of other gliflozin types such as ipragliflozin, tofogliflozin, sergliflozin, remogliflozin, and luseogliflozin. However, regarding the analysis of HF parameters, namely the KCCQ scores, the outcomes from the four analyses, which were considered, although confirmatory of SGLT2i superiority in comparison with placebo, further trials are necessary to elucidate whether these drugs are indicated to improve HF self-declared symptoms evaluated by validated questionnaires as they become routine in clinical practice. Despite its license to be used for HF assessment, it may be more popular and applicable to researchers. The short version of the KCCQ score has not yet been approved for such purposes. Perhaps, a review of this would also be helpful for the scientific community involved in HF research. Ethnicity data was not provided in a few studies [15]. Finally, this study did not assess cardiac parameters such as strain rate. 

Recommendations

Although this systematic review and the multiple studies mentioned in this research have shown the efficacy of SGLT2i in HF treatment, some authors have advocated that the prescription of these drugs is slow owing to the high costs involved in obtaining them [5]. To fill this gap, Iguchi et al. proposed their use as soon as HF symptoms develop, instead of when NYHA IV presentations occur [41]. Hospital discharge after acute HF decompensation admissions represents an optimal timely intervention to offer patients the best EBM [42]. Additionally, considering that HF hospitalizations are high expenditures, owing to the consequences associated with them, if governments subsidized part of the cost or reduced those medication taxes, it would be mutually beneficial for affected patients and institutions.

Moreover, there is scarce literature data on SGLT2i in T1DM, despite vast information about how it might compromise microcirculation over time. To date, only a few short-term and small sample-size studies have evaluated the influence of SGLT2i in these patients. Therefore, multi-centric randomized controlled trials are recommended to explore the benefits to this population, especially when there is also concern about their safety due to genitourinary infection reports in this group of people and other types of gliflozins such as ipragliflozin, tofogliflozin, sergliflozin, remogliflozin, or luseogliflozin, which have not been adequately appraised to the present date [43-45].

Considering that Chagas disease is an underlying cause of CHF, RCTs on SGLT2i in this subset of patients are necessary to recommend this therapy for individuals affected by Chagas disease.

Novel perspectives involving the SGLT2i approach in other heart conditions and diverse medical fields

In addition to what has been previously suggested, the cardioprotective role of SGLT2i has been now studied to assess their safeguarding action against cardiotoxic agents that are employed in chemotherapy to treat cancer, particularly anti-tumor antibiotics (anthracyclines), cell death stimulators (proteasome blockers), and cell growth inhibitors (tyrosine kinase stoppers). A few studies have reported that SGLT2i can mitigate heart toxicity induced by chemotherapeutic drugs [46,47]. Similarly, Dabour et al. stated in their review that some in vivo studies have depicted advantageous results with respect to SGLT2i protection against anticancer therapeutics, thus representing a powerful arsenal in the cardio-oncology field [48].

Likewise, SGLT2i exerted beneficial effects in terms of amending diabetic retinopathy in comparison to other hypoglycemic drugs, presumably under the attenuated release of reactive oxygen species within the retinal vascular bed [49]. Conversely, a meta-analysis by Zhou et al. showed no meaningful causal effect between SGLT2i and ophthalmological disorders in T2DM patients, except for dapagliflozin. In contrast, according to the authors, empagliflozin may slow retinopathy progression [50].

In addition, the Ertugliflozin for Functional Mitral Regurgitation Associated with Heart Failure: EFFORT Trial published by Kang et al. included 128 randomly assigned patients to receive either ertugliflozin or placebo and revealed satisfactory results supportive of SGLT2 inhibition regarding the management of HFpEF and HFmrEF and respective EF ranging from 35% to 50% associated with mitral regurgitation. Nonetheless, further reproducible trials are necessary to ensure the external validity of those findings [51].

Recent in vitro evidence has suggested that SGLT2i may impede different liver cell lineage proliferation, while animal and human studies point out their efficacy against non-alcoholic fatty liver disease (NAFLD) through mechanisms that go beyond the weight loss previously stated [7,52]. Yen et al., in a retrospective cohort analysis, discovered that SGLT2i amend nephritis caused by systemic lupus erythematosus in patients who also present T2DM when compared to placebo. In addition, those who took SGLT2i showed a diminished probability of renal replacement therapy (either by dialysis or transplant) as well as HF events and death from any cause [53].

Considering rheumatologic diseases, SGLT2i displayed valuable superiority compared with placebo in attenuating gout exacerbation frequency and reduced mortality rate in T2DM patients [54]. In a pilot study by Cannarella et al., T2DM male patients expressed improvement in erectile dysfunction after the initiation of dapagliflozin [55].

## Conclusions

This systematic review contributed to the scientific literature by endorsing SGLT2i as impressive cardiorenal protective agents against hospitalization for HF, MACE, progression of renal disease, and self-reported amelioration of KCCQ scales. Based on these findings, SGLT2i may be considered an adjunctive therapy in the HF approach. Such results will also help investigators elucidate the pathophysiology of HF and contribute to the development of other therapeutic options. Individuals affected by the condition may benefit from affordable alternatives that bring the quality of life to and attenuate the whole burden with it, be it a person-to-person interaction or mediated by institutional agents such as hospitals or governmental tax-collector representatives. Recent data related to the efficacy of gliflozins in other medical fields have also granted SGLT2i a groundbreaking position as a pleiotropic drug in the 21st century, similar to what happened to statins in previous decades. Finally, it is noteworthy that, despite premature positive analysis related to the benefits in cardio-oncology, ophthalmology, and other medical specialties, a greater body of evidence composed of RCTs is necessary to corroborate these presumptions.
